# Osteogenesis Modulation: Induction of Mandibular Bone Growth in Adults by Electrical Field for Aesthetic Purposes

**DOI:** 10.1007/s00266-021-02600-0

**Published:** 2021-10-07

**Authors:** Gregorio Hernandez Zendejas, Marek K. Dobke, Andrew Phelps, Gabriel Planas, Marco Sanchez

**Affiliations:** 1 University of California Medical Center, 200 West Arbor Drive, San Diego, CA 92103-8890 USA; 2 Jalisco Institute for Reconstructive Plastic Surgery, 2022 Federalismo Norte, 44220 Guadalajara, Jalisco Mexico

**Keywords:** Facial implant, Mandibular hypoplasia, Microgenia, Genioplasty, Osteogenesis, Osteotomy

## Abstract

**Background:**

A new technique in plastic surgery termed Osteogenesis Modulation is described. This technique uses a surgically implanted, battery-operated medical device to deliver customized electrical pulses to produce mandibular bone growth. This device was designed to be a temporary, nonpermanent implant. The purpose of this study was to review both the safety and efficacy of Osteogenesis Modulation.

**Methods:**

This study comprises two phases. Phase I involved experimental technology development and animal experiments. Phase II included technology development for clinical use and a clinical trial. In Phase II, four patients with a diagnosis of mandibular hypoplasia and microgenia underwent surgical implantation of the novel medical device over the chin bone. Once a satisfactory change of contour of mandibular bone was achieved, the devices were removed. In all patients, the devices were left in place for 12 months, then surgically removed under local anesthesia. Preoperative and long-term postoperative cephalometric controls were done.

**Results:**

In all patients, symmetrical mandibular bone growth was observed with good-to-excellent aesthetic results. The overall follow-up period was 39 months. Cephalometric controls taken 3 to 6 months after the device removal showed an average increase in mandible length of 5.26mm (range, 2.83–7.60mm)

**Conclusions:**

Preliminary clinical results suggest that Osteogenesis Modulation is a safe, minimally invasive, and effective alternative treatment for the correction of mandibular hypoplasia in selected cases.

**Level of Evidence IV:**

This journal requires that authors assign a level of evidence to each article. For a full description of these Evidence-Based Medicine ratings, please refer to the Table of Contents or the online Instructions to Authors www.springer.com/00266.

## Introduction

Mandibular hypoplasia and microgenia continue to be common facial skeleton deformities. The treatment of mandibular hypoplasia and microgenia involves either osteotomies, bilateral sagittal split osteotomy (BSSO), genioplasty, distraction osteogenesis, or alloplastic implants [1–7]. Osteotomies, BSSO, genioplasty, and distraction osteogenesis require general anesthesia and a long postoperative recovery. Alloplastic implants are utilized for aesthetic improvement of the facial profile. However, alloplastic implants may cause underlying bone resorption and foreign body reaction [8, 9]. Resorbable tissue fillers offer a minimally invasive, but temporary solution.

Bone formation and resorption is a dynamic lifelong process. Multiple factors influence bone formation and its remodeling. Recognizing the potential of piezoelectric effect on bone described by some authors [10–24] we developed a new device and technique to produce mandibular bone growth. The purpose of this study was to achieve correction of mandibular hypoplasia and microgenia without using osteotomies, BSSO, genioplasty, distraction osteogenesis, alloplastic implants, or tissue fillers. This study comprises two phases. Phase I involved experimental technology development and animal experiments. Phase II included technology development for clinical use and a clinical trial.

## Materials and Methods

### Phase I: Animal Experiments

**Fig. 1:**
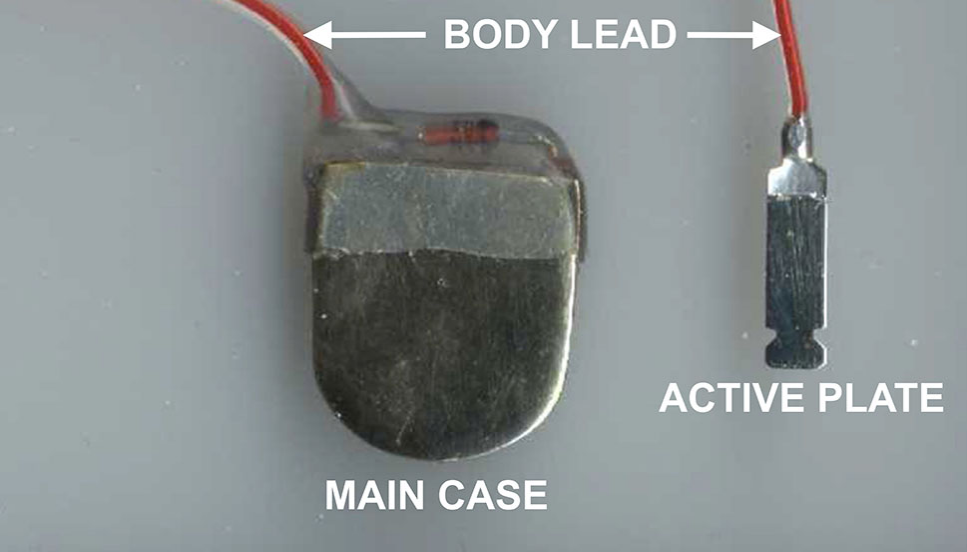
The experimental pulse generator. The generator comprises a platinum-coated main case containing circuitry and a battery. This case is connected to a platinum active plate through a silicone-coated lead. The electric pulses are delivered by the active plate

From June 1999 to March 2001, an experimental study was performed. The study involved four 7-week-old Wistar rats. To elucidate the biological effects of various types of electrical pulses on the mandibles of rats, we developed an experimental, miniaturized, implantable pulse generator (Fig. 1). This experimental device was designed, engineered, and assembled by the first author (G.H.Z.) assisted by a small team of engineers. This device was designed to be a temporary, nonpermanent implant. The experimental pulse generator technical specifications are as follows: The generator comprises a platinum-coated main case containing circuitry and a battery. The main case outer dimensions are 15.0 x 13.0 x 3.5 mm. This case is connected to a platinum active plate through a silicone-coated lead. The electric pulses are delivered by the active plate. The dimensions of the active plate are 11.0 x 3.0 x 0.6 mm. The generator may include one or two active plates. The generator can produce pulses with variable polarity mode, waveform, pulse frequency, pulse amplitude, and pulse width.

For this experimental study, we built three generator models as follows:

1. The first generator comprised one active plate delivering negative polarity pulses with the following characteristics: Rectangular waveform, frequency = 0.5 Hz, pulse amplitude = −720 millivolt, pulse width = 4 milliseconds.
2. The second generator comprised one active plate delivering negative polarity pulses with the following characteristics: Rectangular waveform, frequency = 0.5 Hz, pulse amplitude = −1180 millivolt, pulse width = 4 milliseconds.
3. The third generator comprised two active plates. The first plate of the third generator delivered negative polarity pulses with the following characteristics: Rectangular waveform, frequency = 0.5 Hz, pulse amplitude = −1360 millivolt, pulse width = 4 milliseconds. The second plate of the third generator delivered positive polarity pulses with the following characteristics: Rectangular waveform, frequency = 0.5 Hz, pulse amplitude = +1360 millivolt, pulse width = 4 milliseconds.

**Fig. 2:**
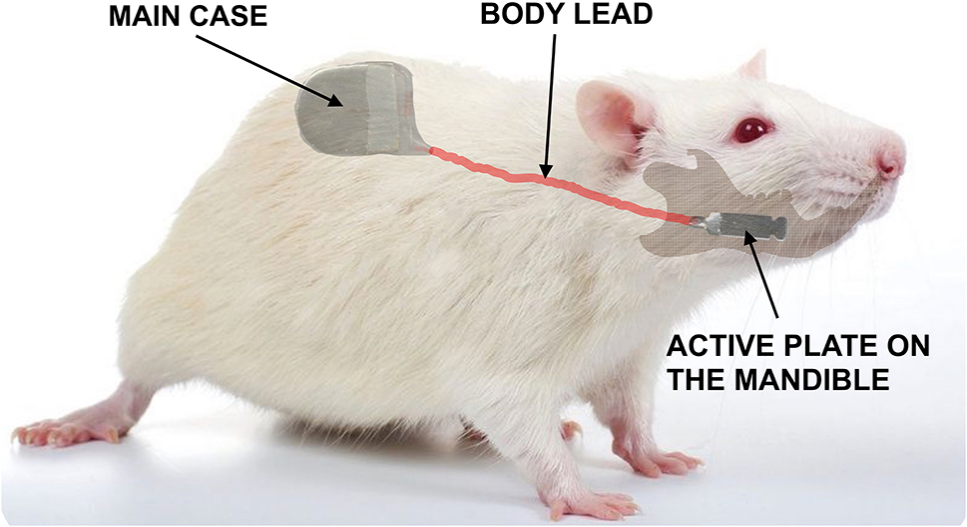
Schematic representation of the subcutaneous location of the experimental pulse generator

The implantation of the experimental pulse generator was carried out as follows (Fig. 2): Under general anesthesia, an incision was made on the rat’s back. A second small incision was made on the skin over the mandible body. The device was inserted through the rat’s back incision, and the active plate was passed subcutaneously to the second incision area. Then, the plate was applied over the mandibular body and secured in place using a nylon 6-0 stitch. The skin was closed using 6-0 nylon.

To elucidate the biological effects of various types of electrical pulses on the mandibles of the rats, we allocated the generators as follows:

1. First rat: No generator was implanted. A simple platinum plate was applied to the body of the left-side mandible. The right-side mandible was left intact.
2. Second rat: The first generator was implanted. An active plate delivering negative polarity pulses of low intensity (−720 millivolt) was applied to the body of the left-side mandible. The right-side mandible was left intact.
3. Third rat: The second generator was implanted. An active plate delivering negative polarity pulses of moderate-intensity (−1180 millivolt) was applied to the body of the left-side mandible. The right-side mandible was left intact.
4. Fourth rat: The third generator was implanted. An active plate delivering negative polarity pulses of high intensity (−1360 millivolt) was applied to the body of the left-side mandible. An active plate delivering positive polarity pulses of high intensity (+1360 millivolt) was applied to the body of the right-side mandible.

The pulse generators were left in place for four weeks, then the rats were euthanized. All mandibles were resected, cleaned from soft tissue, dried, and subjected to a three-dimensional analysis.

### Results

**Fig. 3:**
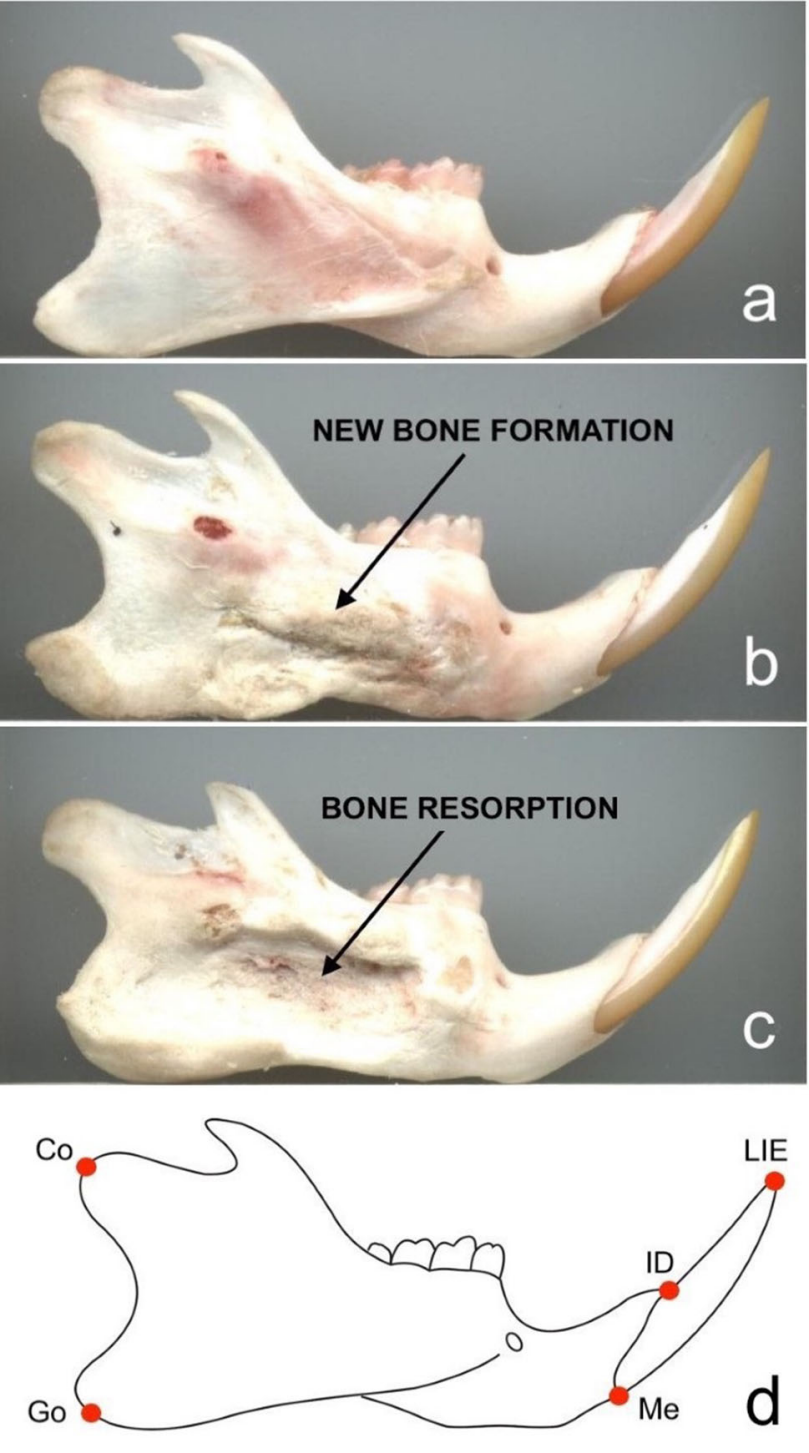
Comparison of the effects of electrical pulses in the rat mandible. **a** Mandible where no pulses were applied. **b** Mandible where negative polarity pulses were applied. New bone formation is evident in the area of application of the active plate. **c** Mandible where positive polarity pulses were applied. Bone resorption is apparent in the area of application of the active plate. **d** Schematic diagram showing the skeletal landmarks used in the three-dimensional analysis of the rat mandibles. Co, condylion; Go, gonion; Me, menton; ID, infradentale; LIE, lower incisal edge. Note: the original picture (b) was flipped horizontally for didactic purposes

No noticeable changes occurred when a single platinum plate was applied to the mandibular body. The negative polarity pulses promoted the formation of new bone, whereas the positive polarity pulses promoted the resorption of bone. A three-dimensional analysis of the rat mandibles was carried out (Fig. 3). An increase of the mandibular thickness directly related to the intensity of the electrical pulses of negative polarity was observed. The average thickness of the control mandibles was 2.64mm (range 2.60–2.66mm). The mandible thickness when low-intensity, moderate-intensity, or high-intensity negative pulses were applied was 2.70mm, 3.43mm, and 3.58mm, respectively. The thickness of the mandible on high-intensity positive pulses was 2.15mm.

Changes in bone remodeling observed at the site of application of the active plate resulted in the overall measured dimensions of the mandible reflecting the fact that “local” changes may affect dentofacial measurements beyond the area of the treatment. The mean Co-Go distance of the control mandibles was 10.62 mm (range 10.28–10.82 mm). The Co-Go distance when high-intensity negative pulses or high-intensity positive pulses were applied was 10.93 mm and 10.53 mm, respectively. The mean Co-LIE distance of the control mandibles was 29.56 mm (range 29.36–29.92 mm). The Co-LIE distance when high-intensity negative pulses or high-intensity positive pulses were applied was 29.79 mm and 29.53 mm, respectively. The mean Co-Me distance of the control mandibles was 23.02 mm (range 22.76–23.34 mm). The Co-Me distance when high-intensity negative pulses or high-intensity positive pulses were applied was 23.03 mm and 23.06 mm, respectively.

### Phase II: Clinical Experience

**Fig. 4:**
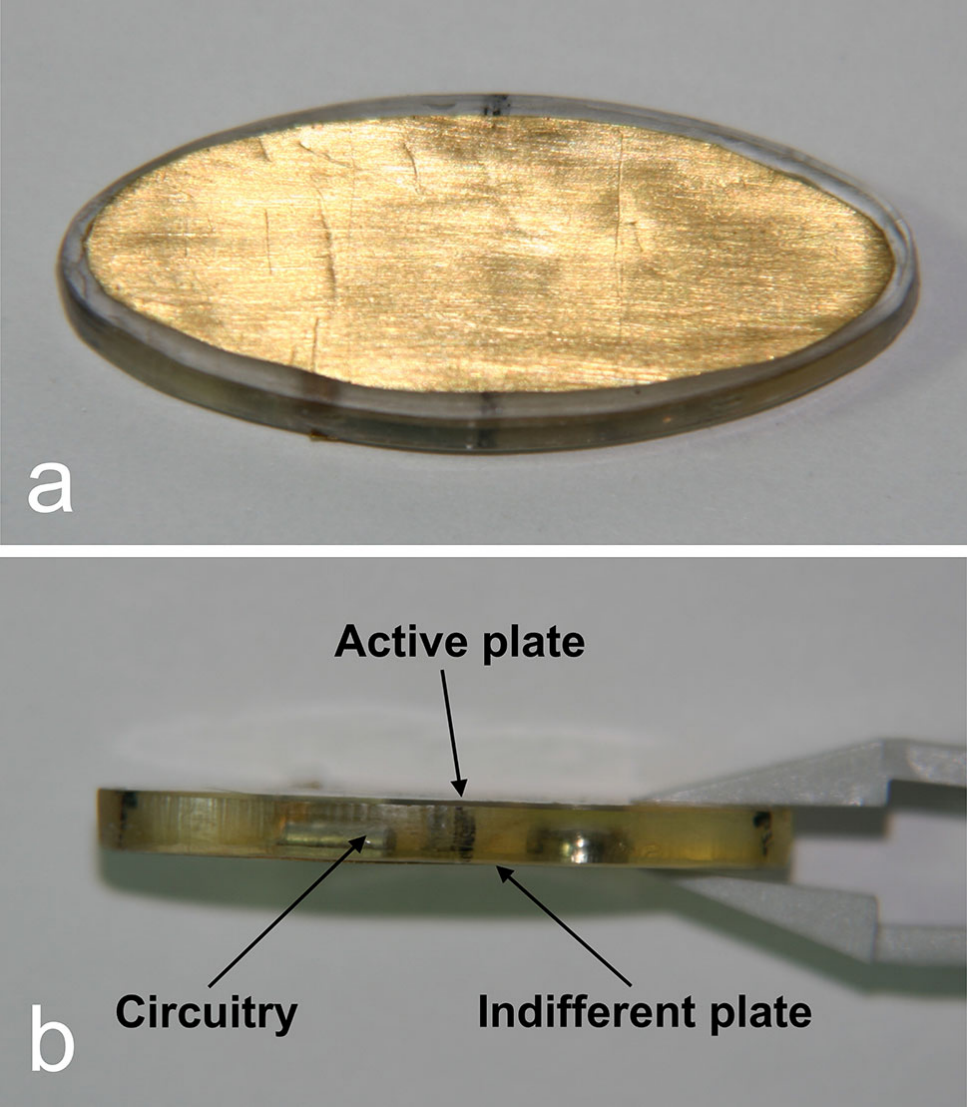
The Modulator. **a** Top view. **b** Side view

From August 2003 to November 2006, a prospective, preliminary observational clinical trial was performed. Patient selection was limited to patients with mandibular hypoplasia and microgenia with normal occlusion. Four patients with a diagnosis of mandibular hypoplasia and microgenia (3 women and 1 man) with a mean age of 20 years (range, 15–25 years) were included. Based on the above-mentioned experimental study, we developed a new device and technique for clinical use. We called this new device “Osteogenesis Modulator Implant” (Modulator, for short), and the new technique “Osteogenesis Modulation” (Modulation, for short) (Fig. 4). The Modulator was designed, engineered, and assembled by the first author (G.H.Z.) assisted by a team of engineers. This device was designed to be a temporary, biocompatible, nonpermanent implant. This device is hermetically sealed and can operate continuously for 18 months. The Modulator technical specifications are as follows: The device dimensions are 30.0 x 13.0 x 3.0 mm. The Modulator comprises a medical grade polymethyl methacrylate case containing miniaturized high-performance circuitry and a battery. Attached to each of its flat surfaces, are 0.1 mm thick medical grade gold plates, which are connected to the circuitry. One of the plates is the active plate, whereas the other is the indifferent plate. The active plate delivers negative polarity pulses with the following basic characteristics: Rectangular waveform, frequency = 4 Hz, pulse amplitude = −1000 millivolt, pulse width = 1.5 milliseconds. Some sterilized Modulator prototypes were built between July 2001 and June 2003.

The surgical technique for the implantation of the Modulator is similar to the technique for the insertion of an alloplastic chin implant. The surgical technique for the implantation of the Modulator is as follows: The midline of the chin area is marked as a reference point. The proposed location of the Modulator is marked with indelible ink. Under local anesthesia with sedation, the chin is accessed through a submental incision. A subperiosteal pocket is developed to accommodate the Modulator. This pocket should be of appropriate size and shape to allow an easy and precise Modulator placement. The Modulator is inserted using special soft-touch, electrically insulated instruments to avoid device damage. During surgery, it is of uppermost importance to verify that the active plate is the one in contact with the bone. The active plate must be in contact with the chin bone to apply the electrical pulses to produce growth of the mandibular bone. During the immediate postoperative period, the patients reported a “bouncing” sensation in the chin. This sensation disappeared after few days.

**Fig. 5:**
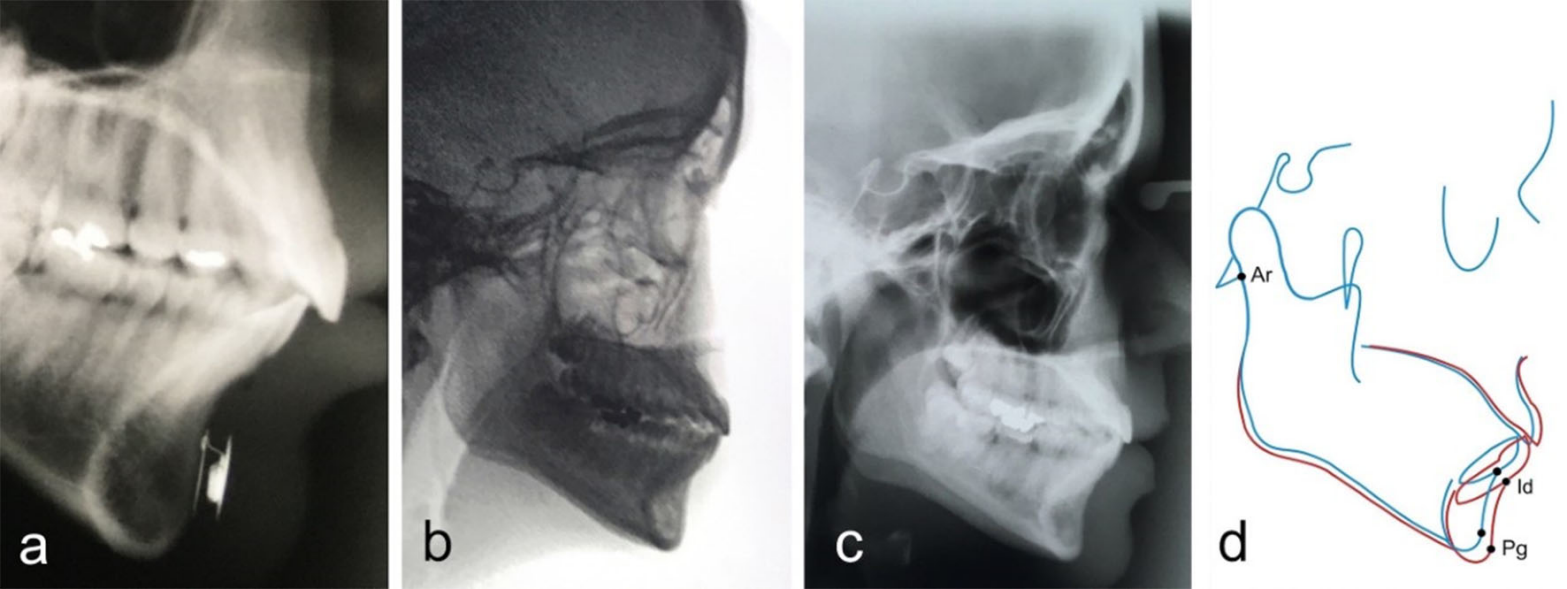
**a** Postoperative X-ray showing the Modulator properly located over the chin bone. The active plate is in contact with the chin bone. **b** Example of a preoperative cephalometric radiograph. **c** Long-term postoperative cephalometric radiograph of the same patient, taken 6 months after the removal of the Modulator. **d** Comparison between preoperative (*blue color*) and long-term postoperative (*red color*) traced cephalometric radiographs of this patient, showing a 7.60mm increase of mandible length and an approximately 3mm increase of the length of the vertical curve between pogonion and infradentale. Ar, articulare (junction between the inferior surface of the cranial base and the posterior border of the ascending ramus of the mandible); Pg, pogonion (most anterior point of mandibular symphysis); Id, infradentale.

**Fig. 6:**
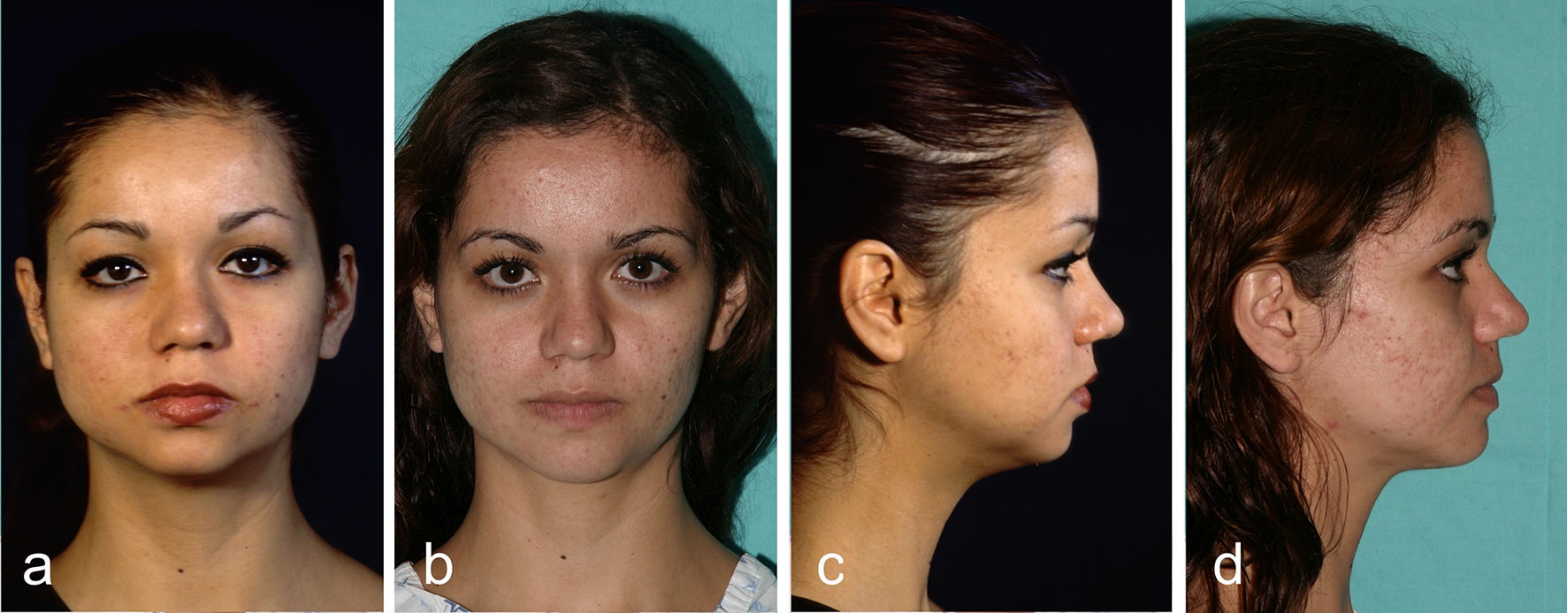
This 19-year-old woman underwent Modulation, and a bichectomy procedure. **a**, **c** Preoperative view. **b**, **d** Postoperative view after 18 months.

**Fig. 7:**
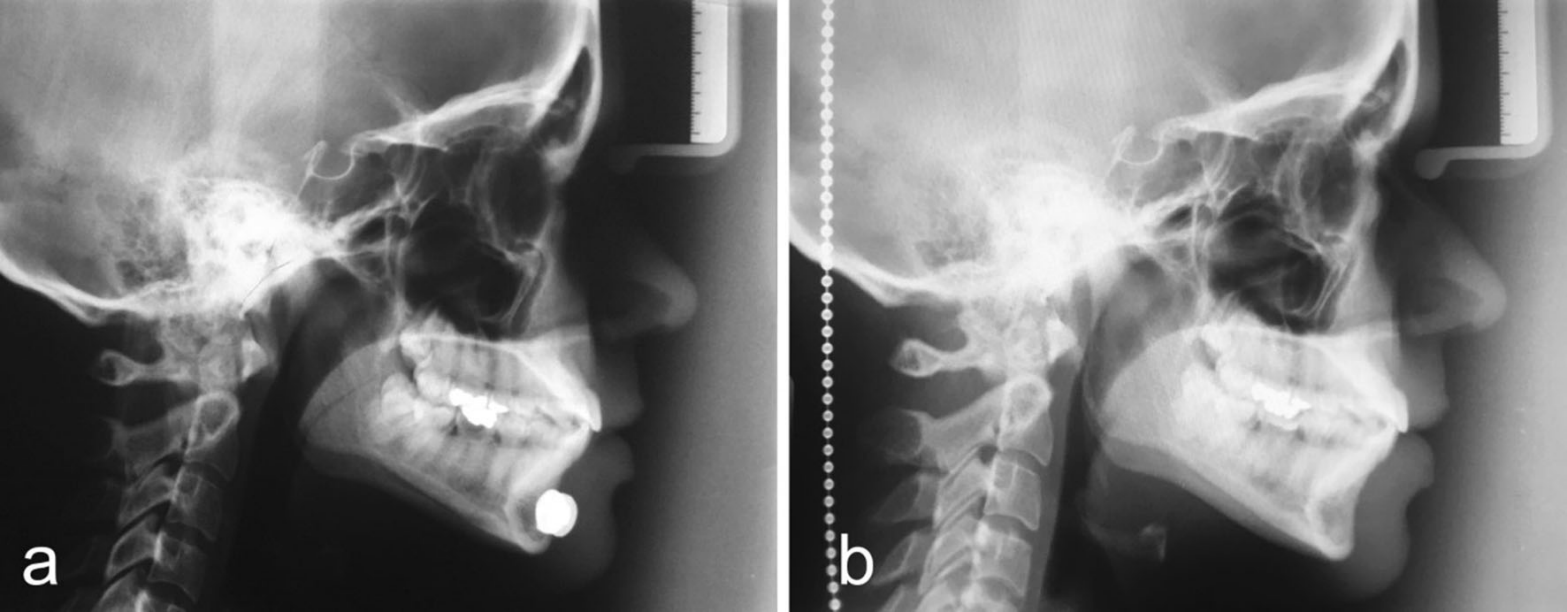
The cephalometric control (**a**—image obtained at the beginning of the treatment and **b**—six months after the Modulator removal) showed a 7.60mm overall length increase of her mandible. Full correction of the mandibular hypoplasia is evident. The change in contour is striking, not only in the body and angle of the mandible but also in the chin area.

**Fig. 8:**
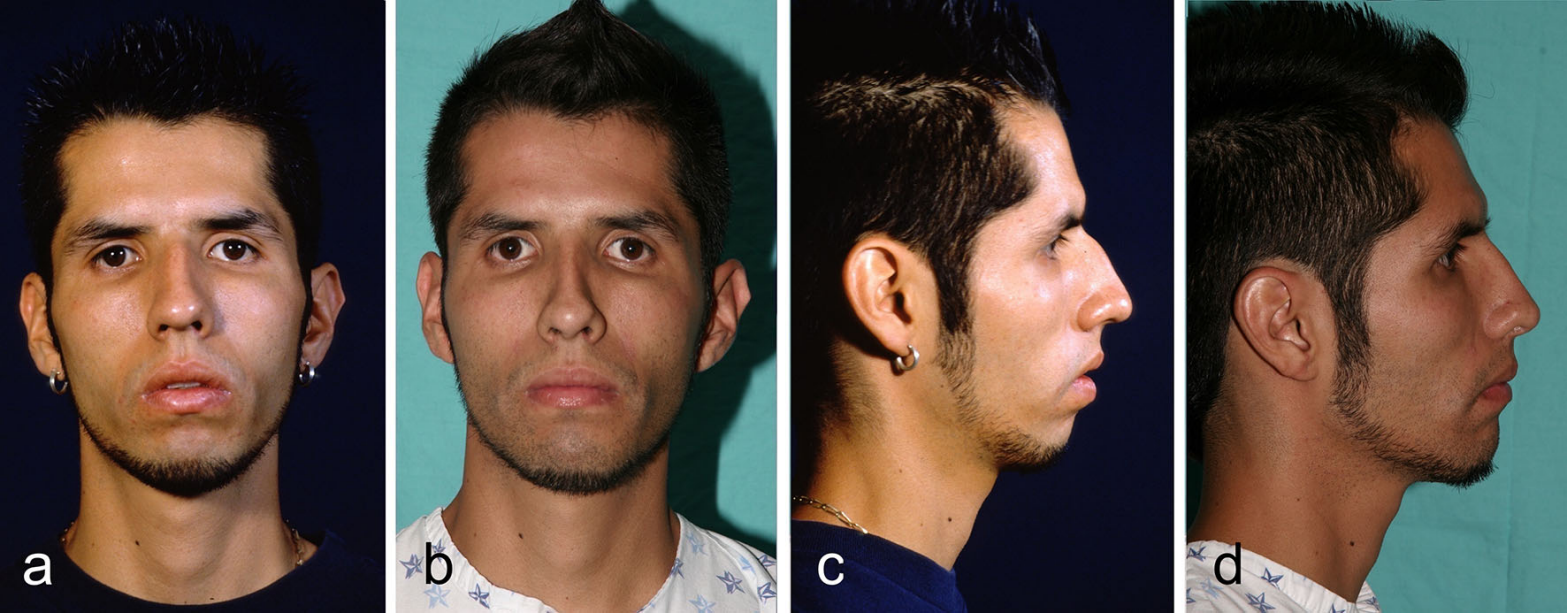
Twenty-one year-old man underwent Modulation, and a rhinoplasty procedure. **a**, **c** Preoperative view. **b**, **d** Postoperative view after 18 months.

**Fig. 9:**
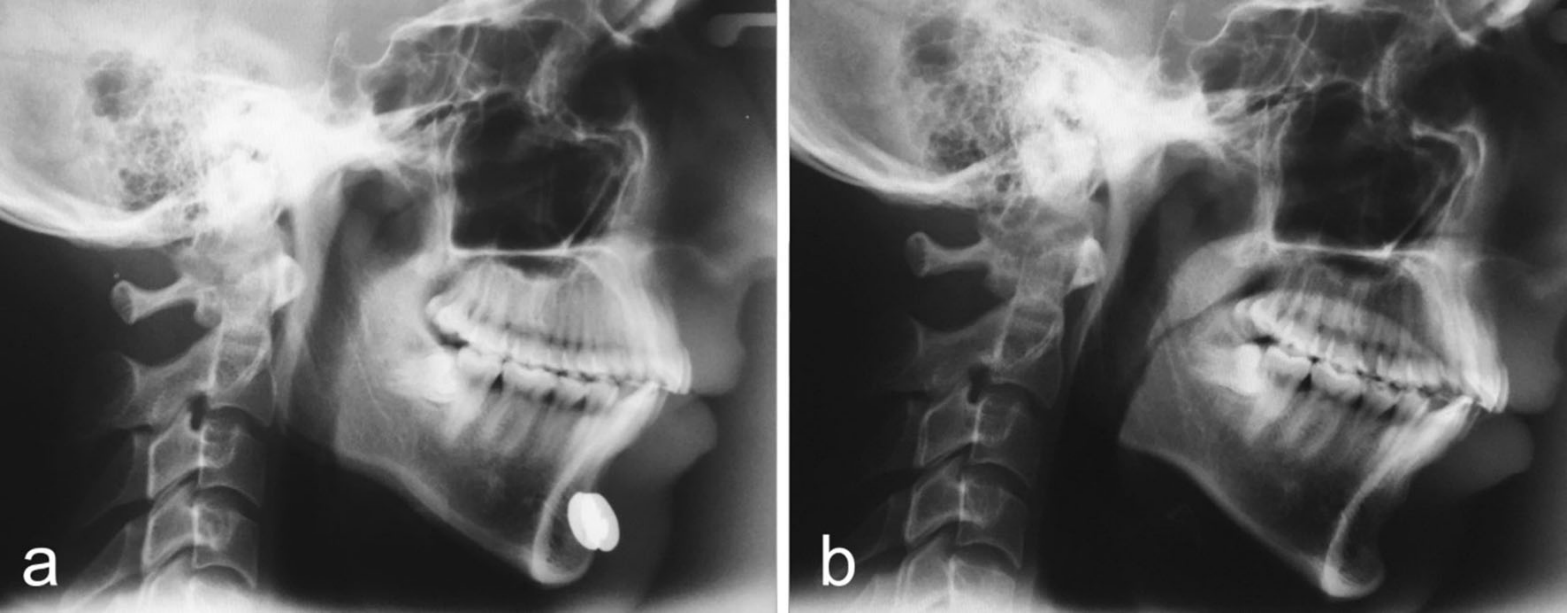
The cephalometric control (**a**—at the beginning of the treatment and **b**—six months after the Modulator removal) showed a 4.85mm overall length increase of his mandible. Correction of the mandibular hypoplasia is apparent. The change in contour is evident, not only in the ramus and angle of the mandible, but also in the body of the mandible, and chin area.

**Fig. 10:**
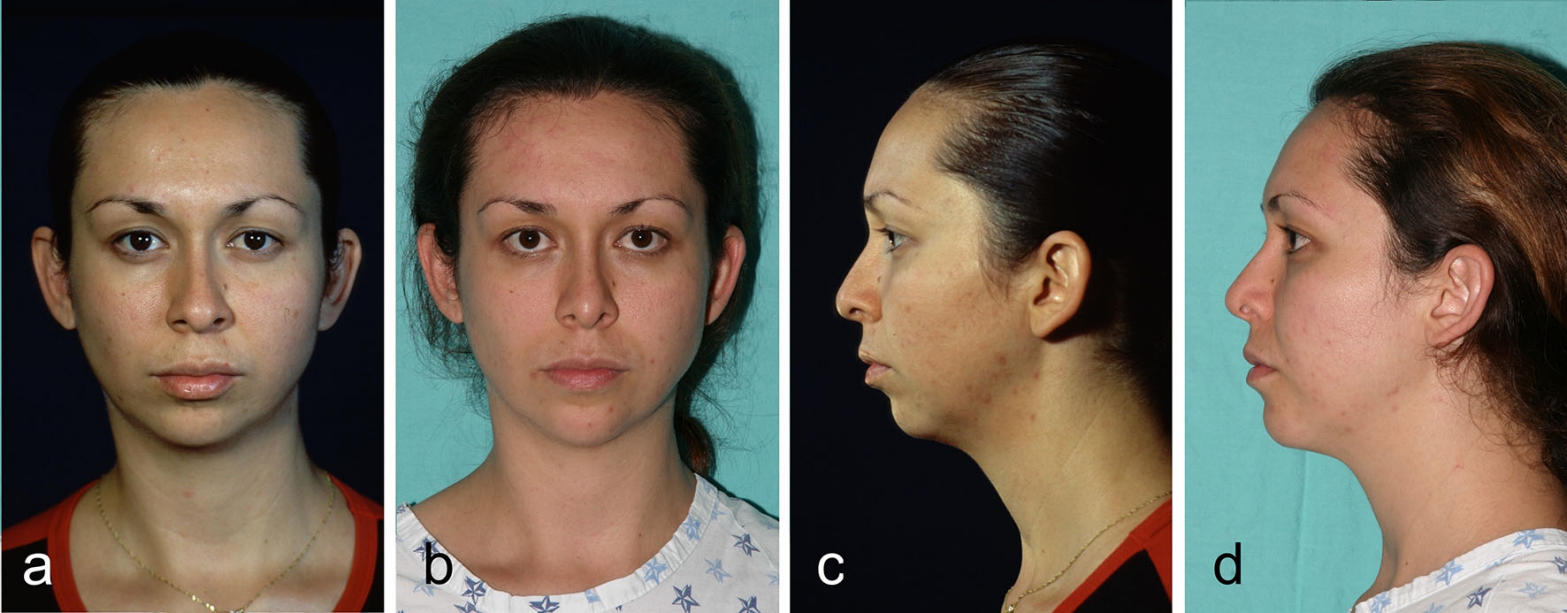
Twenty-five year-old woman underwent Modulation, and a rhinoplasty procedure. **a**, **c** Preoperative view. **b**, **d** Postoperative view after 18 months.

**Fig 11:**
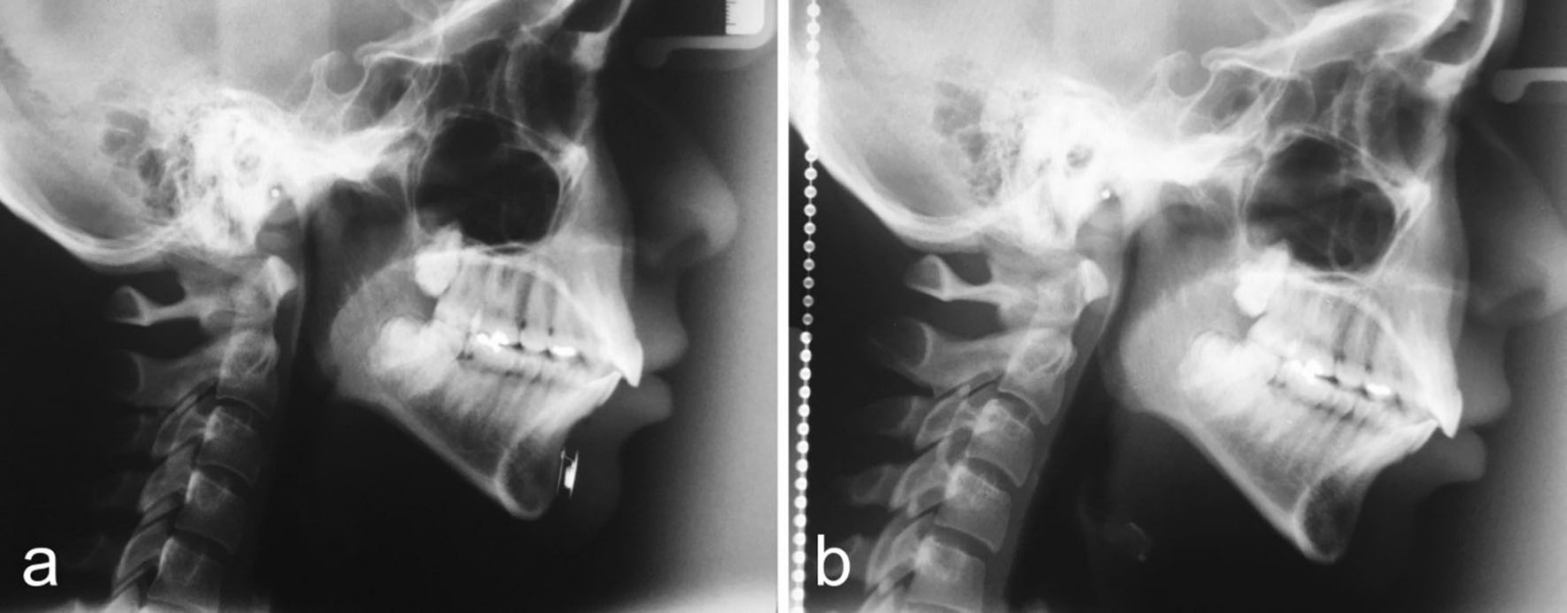
The cephalometric control (**a**—at the beginning of the treatment and **b**—6 months after the Modulator removal) showed a 2.83mm overall length increase of her mandible. The change in contour is noticeable, not only in the body of the mandible but also in the chin area.

**Fig. 12:**
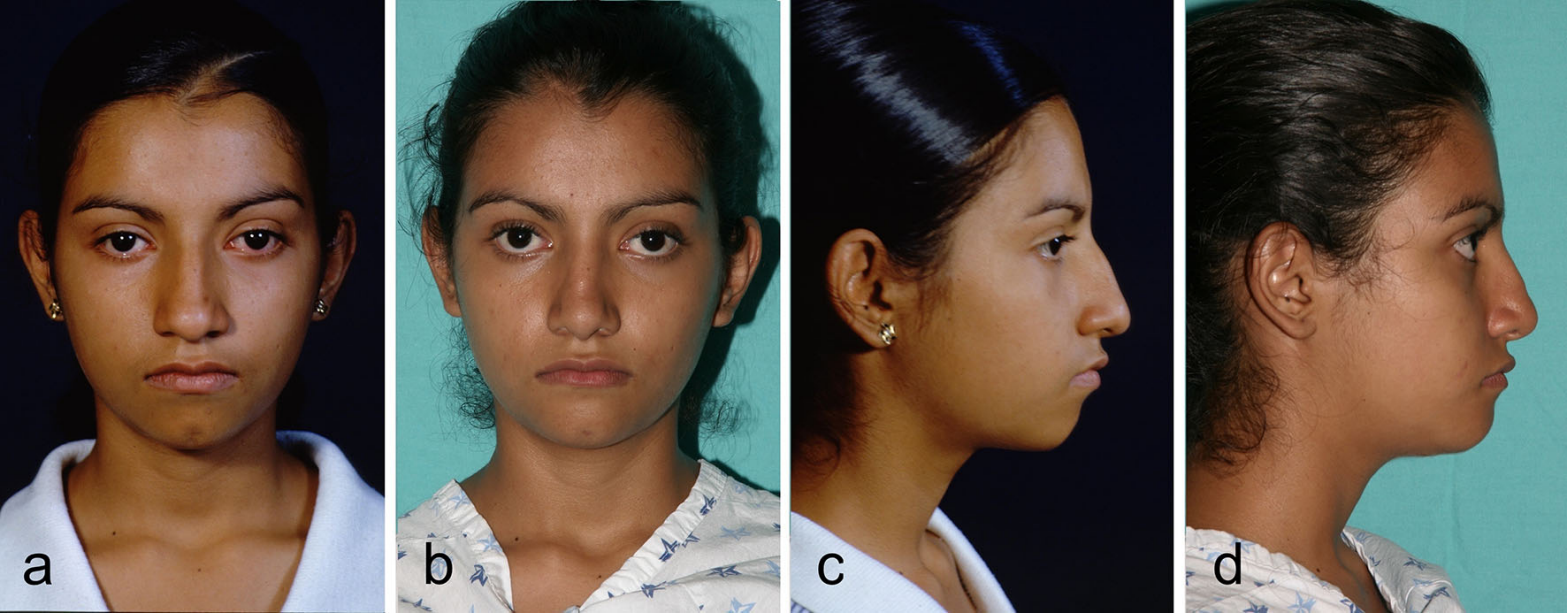
Fifteen-year-old woman underwent Modulation, and a rhinoplasty procedure. **a**, **c** Preoperative view. **b**, **d** Postoperative view after 18 months.

**Fig 13:**
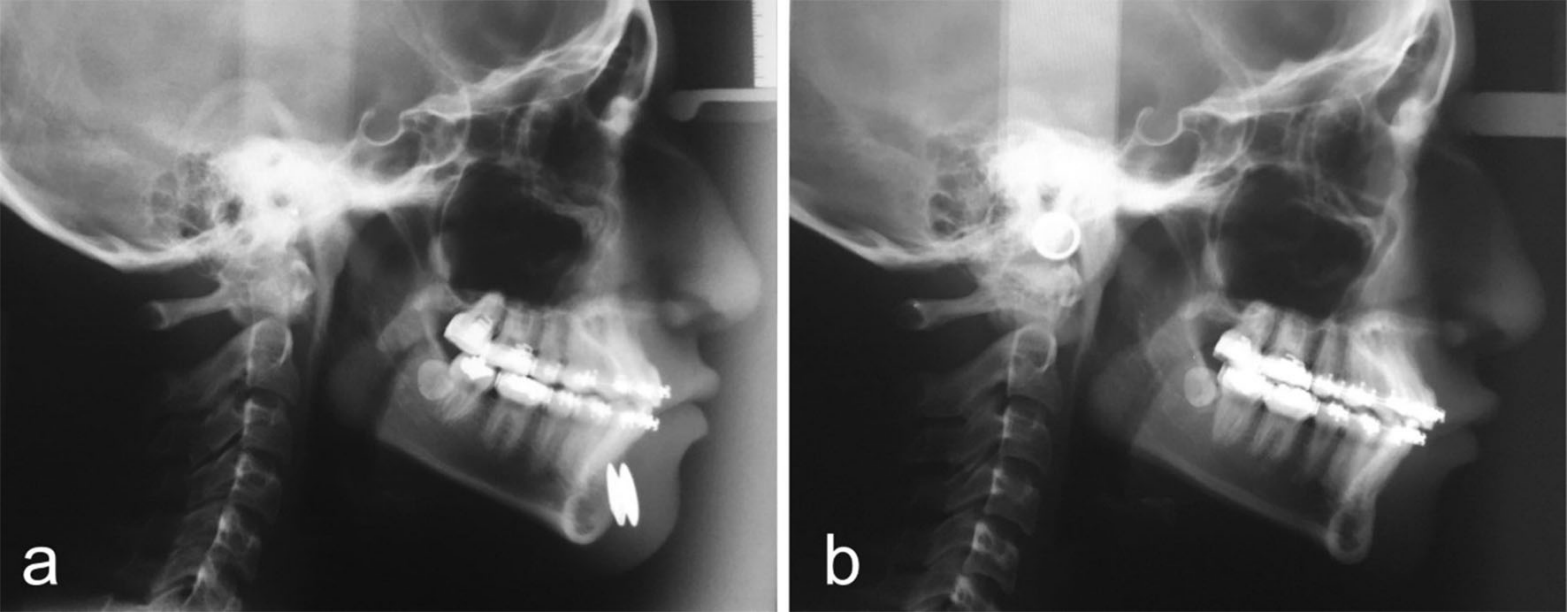
The cephalometric control (**a**—at the beginning and **b**—six months after the Modulator removal) showed a 5.76mm overall length increase of her mandible. The change in contour is noticeable, not only in the body and angle of the mandible but also in the chin area.

Few weeks after surgery, a postoperative X-ray was taken to verify the adequate location of the Modulator. Cephalometric controls were carried out before surgery and six months after the removal of the modulator (Figs. 5, 6, 7, 8, 9, 10, 11, 12, 13). The cephalometric analysis compared the preoperative and the long-term postoperative changes in the distances between articulare (Ar) and pogonion (Pg) and between pogonion and infredentale (Id) cephalometric landmarks. The radiographs were also assessed for net soft tissue advancement by tracing and measuring soft tissue thickness from bony pogonion to soft tissue pogonion [25]. The postoperative follow-up included periodic monitoring of the Modulator function. This monitoring was done using an electrocardiograph (ECG), putting the ECG cutaneous electrodes over the chin area to detect the Modulator electric pulses. In all patients, the Modulators were left in place for 12 months, then surgically removed under local anesthesia.

### Results

To evaluate the changes of the mandible length, we compared the preoperative and the long-term postoperative changes in the distances between Ar and Pg cephalometric landmarks. To assess chin vertical curve changes, the preoperative and postoperative distance between Id and Pg was measured. Good-to-excellent aesthetic results were achieved in all cases. The overall follow-up period was 39 months. Cephalometric controls taken 3 to 6 months after Modulator removal showed an average increase in mandible length of 5.26mm (range, 2.83–7.60mm) and of vertical curve of 23.5mm (range from 22 to 25mm), (Figs. 5, 6, 7, 8, 9, 10, 11, 12, 13). The average increase in soft tissue projection was 90% of the thickness of “added” bone length 4.7 mm (range, 2.5–6.8mm). After 39 months, clinical results were maintained, with no clinical evidence of bone resorption or complications. All patients preserved a normal pretreatment occlusion.

## Discussion

Principles of “tissue engineering” applied to programmable or controllable osteogenesis are attained by the Modulator. The device retains its physical properties after implantation and functions well fulfilling biological expectations [26, 27]. The piezoelectric effect is the capacity of some materials to generate an electric charge in response to applied mechanical stress. Bone exhibits the piezoelectric effect, generating electrical potentials in response to mechanical stress [10, 11]. When the facial bones are subjected to the stress of muscular forces, electrical charges of different polarities are generated. Negative polarity charges promote bone formation, while positive polarity charges promote bone resorption [12–14]. Although there are several experimental reports about the effects of electrical stimulation on osteogenesis and bone remodeling [15–24, 28–53], the Modulator is the world’s first practical, clinical facial implant capable of producing long-term, reliable and sustainable bone remodeling in intramembranous type of bone. In addition to permanent bone augmentation effect, osteogenesis was complemented by soft tissue changes. Similar, if not better outcome, comparing to results produced by implants made of hard materials, soft tissue projection was of approximately 90% of new bone thickness [25].

Modulator implantation procedure requires both a comprehensive knowledge of facial anatomy and some basic training in the use of the special instruments used to insert the Modulator. The surgical technique for the insertion of the Modulator is similar to the technique for the insertion of an alloplastic chin implant. Preliminary clinical results suggest that Modulation is indicated for patients with mandibular hypoplasia and microgenia with normal occlusion. Modulation can be an alternative to tissue fillers, alloplastic implants, mandibular osteotomies, BSSO, genioplasty, and distraction osteogenesis in selected clinical cases. BSSO, osteotomies, genioplasty, and distraction osteogenesis require general anesthesia, have a long postoperative recovery and they have their risk of postoperative complications. On the other hand, Modulation requires local anesthesia with sedation, have a fast recovery period, and a low risk of postoperative complications. Alloplastic implants are useful for aesthetic improvement of the facial profile, require local anesthesia with sedation, and have a fast recovery period. However, alloplastic implants are permanent implants and eventually may cause underlying bone resorption and foreign body reaction [8, 9]. On the other hand, Modulation utilizes a Modulator, which is a temporary implant that is removed once a satisfactory change of facial profile is achieved. Continued investigations will include the 3D analysis (Vectra Technology, Canfield Scientific, Parsippany, NJ) of soft tissue response to placement as well as removal of the Modulator on the overall facial aesthetics [28, 54–56]. Resorbable tissue fillers offer an office-based, temporary solution for aesthetic improvement of the facial profile while Modulation offers a long-term correction of mandibular hypoplasia and microgenia. Other, emerging options for bone regeneration or generation include Theradaptive implants. This technology which is investigated for bone regeneration at this time is based application on application of bone morphogenic protein variant (called AMP-2) to guide migrating stem cells into bioactive implants acting as scaffolds to ensure formation of the bone where needed (within boundaries of the implants). However, this technology effective for repair of bone gaps was not tested for bone “additive objective” such as augmentation of otherwise intact, healthy bone [55].

For programmable osteogenesis research, mostly focused on osteoinductive proteins and their delivery systems for repairs on bone defects, confining osteoinductive activity within boundaries of the delivery vehicle is one of the concerns [26, 27, 56]. One of the observed advantages of Modulator include protected or controlled bone generation, limited to the size of the implant. No uncontrolled prolapse of callus or newly formed bone into the platinum-coated device or soft tissue neighboring the implant was seen [57]. Excessive callus—which could be detrimental in aesthetic applications—formation was reported in some circumstances when external electrical stimulation was applied to enhance endochondral bone fractures healing. Protected bone generation just like protected bone regeneration is essential for bony contouring or defects repair in craniofacial surgery either cosmetic or reconstructive, respectively, [57]. Platinum’s biocompatibility makes it ideal for coating for both short- and long-term medical implants, its high integrity causes that platinum surfaces can withstand stress without deformation or cracking [58]. Mechanisms how bone tissue behavior such as possibly cell migration, proliferation, differentiation and apoptosis all leading to controlled osteogenesis in response to electric stimulations and/or to mechanical—non-electrical field generating—implants strains certainly deserve future laboratory and clinical investigations [59, 60].

One disadvantage of Modulation could be the current low availability of the Modulator. Notwithstanding, eventually the Modulator can be mass-produced which will lower the cost similarly to the trajectory of the development other high-tech medical devices. The current limitations of Modulation are that this technique may not be suitable for patients with severe craniofacial deformities.

## Conclusion

Preliminary clinical results suggest that Modulation can be a practical tool to augment bone contours indicated for patients with mandibular hypoplasia and microgenia with normal occlusion. We achieved acceptable long-term correction of mandibular hypoplasia and microgenia without using osteotomies, genioplasty, BSSO, distraction osteogenesis, alloplastic implants, or tissue fillers. Preliminary clinical results suggest that Modulation is a safe, minimally invasive, and effective alternative treatment for the long-term correction of mandibular hypoplasia and microgenia in selected cases. Further applications of Modulation in plastic surgery are currently being tested.

## Declarations

### Animal Experiments

All applicable institutional and national guidelines for the care and use of animals were followed.
